# *Phialemoniopsis limonesiae* sp. nov. causing cutaneous phaeohyphomycosis in an immunosuppressed woman

**DOI:** 10.1080/22221751.2021.1892458

**Published:** 2021-03-09

**Authors:** D. Alvarez Martinez, C. Alberto, A. Riat, C. Schuhler, P. Valladares, B. Ninet, B. Kraak, P. W. Crous, L. W. Hou, L. Toutous Trellu

**Affiliations:** a Division of Dermatology and Venereology, University Hospital of Geneva, Genève, Switzerland; b Division of Laboratory Medicine, Laboratory of Bacteriology, University Hospital of Geneva, Genève, Switzerland; c Division of Infectious Diseases, University Hospital of Geneva, Genève, Switzerland; d Division of Laboratory Medicine, Laboratory of Dermatology, University Hospital of Geneva, Genève, Switzerland; e Westerdijk Fungal Biodiversity Institute, CT Utrecht, Netherlands; f State Key Laboratory of Mycology, Institute of Microbiology, Chinese Academy of Sciences, Beijing, People’s Republic of China

**Keywords:** Bioavailability itraconazole, deep cutaneous phaeohyphomycosis, immunosuppression, opportunistic fungal infections, a new taxon

## Abstract

Rare or opportunistic fungal infections are mostly described in immunosuppressed patients. We present a case of a cutaneous phaeohyphomycosis that developed on the dorsal foot in an immunosuppressed woman suffering from AIDS, caused by a novel *Phialemoniopsis* species. It clinically presented as an indurated violaceous plaque, surmounted by nodules exuding a sero-purulent discharge. A filamentous fungus was isolated from pus and cutaneous biopsy. ITS and LSU sequences phylogenetically resolved the fungus as an unknown species of *Phialemoniopsis*, which is an unresolved family within *Sordariomycetes*. In this study we describe the new species as *Phialemoniopsis limonesiae*, which clusters on a single branch clearly separated from its closest phylogenetic neighbours. This new strain showed low MIC to itraconazole, voriconazole and posaconazole.

## Introduction

Up to 2000 fungal species are being newly described each year [1], several of which are associated with human infections. The genus *Phialemoniopsis* (*Sordariomycetes*) presently contains six species, four of which have been isolated from human fluids, skin or nails [2]. Since the introduction of *Phialemoniopsis* in 2013 [2], a further two species have been added, namely *P. hongkonensis*, from a nodule on a human forearm [3], and *P. endophytica*, the only species thus far isolated from plants [4].

*Phialemoniopsis*, which is a genus of dematiaceous hyphomycetes, can be distinguished from *Phialemonium* by having phialides and adelophialides with collarettes, and the development of sporodochium- or pycnidium-like conidiomata, which are both absent in *Phialemonium s. str*.

In the present communication, we report a case of cutaneous phaeohyphomycosis in an immunosupressed woman caused by a novel *Phialemoniopsis* species described here as *P. limonesiae*.

## Case report

**Figure 1.**
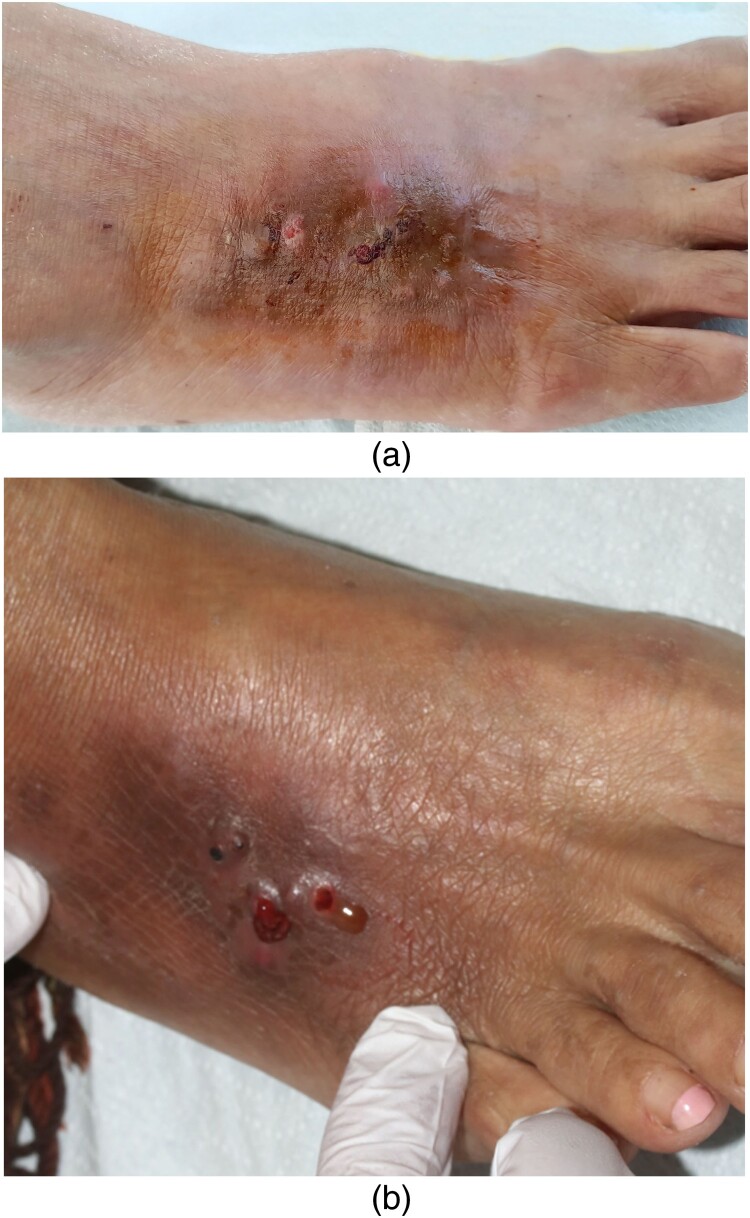
(a) An indurated purplish plaque surmounted by papules and nodules on the dorsum of the right foot, sometimes ulcerated. (b) Violaceous papules and nodules with a sero-purulent discharge.

A 56-year-old woman was referred at our consultation for a persistent wound on her foot. She was followed for AIDS (CD4+ T lymphocytes 38/µl and controlled HIV viremia), disseminated histoplasmosis in 2015, and metastatic pulmonary adenocarcinoma since 2017. Her usual treatment included abacavir/dolutegravir/lamivudine (Triumeq^®^), cotrimoxazole prophylaxis, itraconazole as secondary prophylaxis (200 mg oral capsules bid) since her histoplasmosis and osimertinib 40 mg/kg, respectively. The patient’s history included a trip to Ecuador prior to the onset of the skin lesions. It initially erupted as a unique papule, progressing to multiple violaceous papulo-pustules and local pain (Figure 1(a)). There was no history of skin trauma.

Her general condition was poor with asthenia and anorexia. She had no fever. Skin examination showed an indurated purplish painful plaque surmounted by papules and nodules on the dorsum of the right foot, sometimes ulcerated with a sero-purulent discharge with grains (Figure 1(b)). No peripheral adenopathy was noted. Clinical diagnosis suspected a deep mycosis (including a relapse of histoplasmosis with cutaneous location), bacterial actinomycosis, or mycobacterial infection.

**Figure 2.**
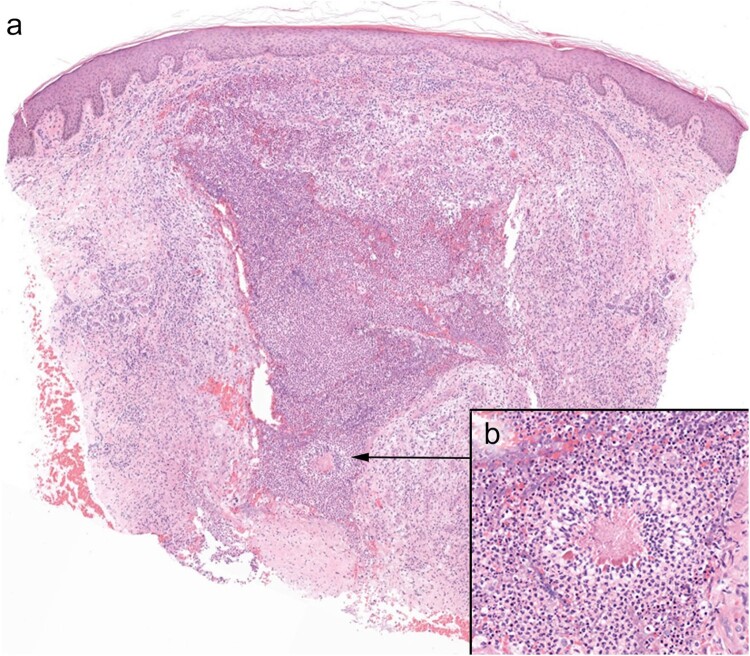
(a) H&E x 40: A dense dermal neutrophilic infiltrate with focal yeast-like structures (2b inset (H&E x 250)).

**Figure 3.**
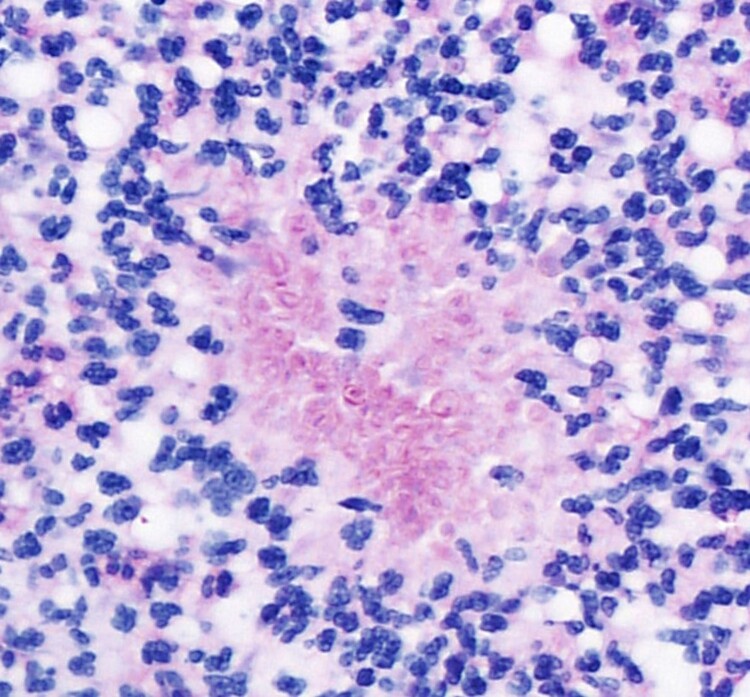
PAS x 400: the use of special stains (PAS) confirmed the fungal nature of the organism.

Histological examination (H&E) revealed a dense dermal, predominantly neutrophilic infiltrate, with abscess formation. The overlying epidermis was slightly acanthotic with parakeratosis and slight spongiosis. Yeast-like structures were seen within the abscess (Figure 2(a,b)). The use of special stains (PAS) confirmed the fungal nature of these structures (Figure 3).

The X-ray did not reveal any signs of osteitis. Bacterial and mycobacterial cultures from swabs and cutaneous biopsy were negative. Two repeated swabs from the pus and one cutaneous biopsy showed, on direct examination, the presence of hyphae which grew on brain heart infusion (BHI) agar with chloramphenicol and gentamicin after 5 days incubation at 30°C, later to be identified as a species of *Phialemoniopsis* by ITS sequencing. The identification by MALDI-TOF MS (Bruker Daltonics) using two different data bases showed a discrepancy. The results obtained with the Bruker database (MBT Compass Library 8431 MSP) and the MSI database were *Phialemoniopsis curvata* (score: 2.03) and *Phialemoniopsis cornearis* (score: 21.29), respectively [3,5]. Cultures from the swab and skin biopsy were sent to the Westerdijk Fungal Biodiversity Institute, in the Netherlands, for species identification*.* The strain was suspected to be a new species of *Phialemoniopsis*, and is described below.

The susceptibility testing (Sensititre^TM^ Yeastone, Thermo Fisher Scientific) (MIC, minimal inhibitory concentrations μg/ml) showed: flucytosine > 64; posaconazole 0.06; voriconazole 0.12; itraconazole 0.06; fluconazole 16; amphotericin B 2.

The clinico-microbiological presentation was consistent with the diagnosis of a deep cutaneous phaeohyphomycosis of the foot in a context of immunosuppression.

We treated the patient with povidone iodine and after culturing the fungus, itraconazole capsules were replaced by an oral solution of itraconazole 200 mg b.i.d. in order to increase the biodisponibility in this HIV+ patient [6]. The lesion improved with plaque regression and diminution of nodule infiltration under itraconazole solution. A relapse was noted when we changed the compounded formulation (solution to capsule) due to adverse effects (nausea). At that time low itraconazole blood level (0.81 mg/ml) was confirmed under capsules intake. In addition, we topically applied a compress soaked with itraconazole solution, twice a day for 10 min. After 5 weeks of oral and topical itraconazole, the cutaneous lesions were stable and asymptomatic. The new itraconazole blood level was 1.40 mg/ml.

The patient died of her oncologic issue after 7 months of treatment. The cutaneous lesions regressed but had not completely healed at the time of her death.

## Material and methods

The new species of *Phialemoniopsis* was described by standard morphological examination of the fungal culture and by molecular phylogenetic analysis.

Strains isolated from both pus and cutaneous biopsy from the patient were inoculated on brain heart infusion (BHI) agar (BD Difco^TM^) with chloramphenicol (50 µg/ml) and gentamicin (50 µg/ml) and on Candida CHROMagar plates (CHROMagar Candida, France). First growth was seen after 5 days of incubation at 30°C.

Total genomic DNA was extracted from fresh mycelia using the Wizard® Genomic DNA Purification Kit (Promega Corporation, Madison, WI, USA), following the manufacturer’s protocol. The internal transcribed spacer (ITS) regions and the 28S rRNA gene (LSU) were amplified and sequenced with the primer pairs ITS5/ITS4 [7] and LR0R/LR5 [8,9], respectively. Fragments of the beta-tubulin genes (*tub2*) and actin (*act*) were amplified with the primer sets TUB4Fd/Bt1b [10,11] and LPW26272/LPW26273 [3]. Polymerase chain reaction (PCR) protocols followed established protocols [12]. The program SeqMan v. 12.1.0 (DNASTAR, Madison, WI, USA) was used to obtain consensus sequences of each isolate. Novel sequences generated in this study were deposited in GenBank (Table 1), and the taxonomic novelties in MycoBank [13, http://www.MycoBank.org]. Sequences of each locus were aligned through MAFFT v. 7 [14], using the default parameters, and were manually corrected in MEGA v. 6.06 [15].

**Table 1.** *Phialemoniopsis* isolates and ex-type or reference strains of related species included in the study.

Isolate	Origin	Species	LSU	*act*	ITS	*tub2*
UTHSC 06-1465	Shin aspirate, USA	*Phialemoniopsis cornearis*	HE599270	HE599253	HE599285	HE599302
UTHSC 06-1820 T	Corneal fluid, USA	*Phialemoniopsis cornearis*	HE599269	HE599252	HE599284	HE599301
UTHSC 04-956	Sinus, USA	*Phialemoniopsis curvata*	HE599278	HE599263	HE599295	HE599312
UTHSC 06-4324	Canine pleural fluid, USA	*Phialemoniopsis curvata*	HE599273	HE599256	HE599288	HE599305
UTHSC 08-2292	Blood, USA	*Phialemoniopsis curvata*	HE599277	HE599262	HE599294	HE599311
UTHSC R-3447	Eye, Israel	*Phialemoniopsis curvata*	HE599274	HE599259	HE599291	HE599308
UTHSC R-3448	Eye, Israel	*Phialemoniopsis curvata*	HE599275	HE599260	HE599292	HE599309
CBS 490.82 T	Skin lesion, USA	*Phialemoniopsis curvata*	FR691977	HE599258	HE599290	HE599307
CBS 491.82	Soil, USA	*Phialemoniopsis curvata*	FR691976	HE599257	HE599289	HE599306
ACCC 38978	*Luffa cylindrica*, China	*Phialemoniopsis endophytica*	KT799558	KT799552	KT799555	KT799561
ACCC 38979	*Luffa cylindrica*, China	*Phialemoniopsis endophytica*	KT799559	KT799553	KT799556	KT799562
ACCC 38980 T	*Luffa cylindrica*, China	*Phialemoniopsis endophytica*	KT799560	KT799554	KT799557	KT799563
HKU39 T	the forearm, China	*Phialemoniopsis hongkongensis*	KJ573447	KJ573452	KJ573442	KJ573457
**CBS 146752 T**	**Genève, Switzerland**	***Phialemoniopsis limonesiae***	**MW050976**	**MW349126**	**MW050977**	**MW048608**
IHEM 19077 T	Keratomycosis, Brazil	*Phialemoniopsis ocularis*	HE599264	HE599247	HE599279	HE599296
UTHSC 07-3736	Left hand, USA	*Phialemoniopsis ocularis*	HE599268	HE599251	HE599283	HE599300
UTHSC 09-2358	Aspirate cellulitis, USA	*Phialemoniopsis ocularis*	HE599267	HE599250	HE599282	HE599299
UTHSC 04-7 T	Toe nail, USA	*Phialemoniopsis pluriloculosa*	HE599271	HE599254	HE599286	HE599303
UTHSC 09-3589	Synovial fluid, USA	*Phialemoniopsis pluriloculosa*	HE599272	HE599255	HE599287	HE599304
CBS 604.67	Noodles, Ukraine	*Phialemonium atrogrise*	HE610470	HE599327	HE610367	HE599346
CBS 279.76 T	Systemic infection, USA	*Phialemonium obovatum*	FR691997	HE599315	HE610365	HE599334

Phylogenetic analyses were based on a concatenated alignment of *act*, ITS, LSU, and *tub2*. The Maximum Likelihood (ML) analyses were performed on the CIPRES Science Gateway portal [16] using RAxML v. 8.2.9. For ML analyses, the default parameters were used, and bootstrap support (MLBS) was carried out using the rapid bootstrapping algorithm with the automatic halt option. A MLBS value >50% was considered as statistically significant. Bayesian analyses (BI) were performed using MrBayes v. 3.2.6 [17]. Markov Chain Monte Carlo sampling (MCMC) analyses of four chains were started in parallel from a random tree topology. Four simultaneous Markov chains were run for 10 M generations with a sampling frequency set to the 1000th generation (resulting in 10,000 total trees per parallel run). The first 25% trees represented the burn-in phase of the analyses and were discarded, and the remaining trees were used to calculate posterior probabilities (BPP). FigTree v. 1.4 was used to visualize the final tree.

Morphological features were determined on oatmeal agar (OA), 2% potato dextrose agar (PDA), 2% malt extract agar (MEA) and synthetic nutrient-poor agar (SNA) [18]. Cultures were incubated at 25°C in the dark for 2 weeks. Macroscopic characters and diameters were measured after 14 d of incubation, and the colony colour (surface and reverse) rated after Rayner (1970) [19]. Slide preparations were mounted in clear lactic acid to study the micromorphological structures. Micromorphological observations were processed with a Nikon Eclipse 80i compound microscope with differential interference contrast (DIC) optics and a Nikon AZ100 dissecting microscope, both equipped with a Nikon DS-Ri2 high-definition colour digital camera. Photomicrographs and measurements were taken with a Nikon DS-Ri2 digital camera using the NIS-elements D software v. 4.50. At least 30 observations (length and width) were made of each structure, and the extremes calculated.

## Results

Twenty-one combined *act*, ITS, LSU, and *tub2* sequences were aligned including *Phialemonium obovatum* CBS 279.76 and *Phialemonium atrogrise* CBS 604.67 as outgroup, comprising 2168 characters including gaps after alignment (501 for ITS; 462 for LSU; 457 for *tub2*, and 748 for *act*). The topology of the BI tree was confirmed by ML analysis for the distinction of nine well-supported monophyletic lineages. The Maximum Likelihood tree based on the combined dataset was presented with the bootstrap support values of the ML analysis (MLBS) and relevant Bayesian posterior probabilities (BPP) shown at the nodes (MLBS > 50%, BPP > 0.80; Figure 5). The strain isolated in this study clustered within the genus *Phialemoniopsis*, but proved to be distinct from any known species (Figure 5).

*Phialemoniopsis limonesiae* A. Riat, L.W. Hou & Crous, *sp. nov.* MycoBank MB837525.

*Etymology*: Named after the patient’s name, “Limones,” from whom this fungus was isolated. An informed consent was obtained from the patient in order to present her case and name this novel fungus after her.

*Typus*: Switzerland, Genève, from a wound on the foot of a women patient, 2019, A. Riat (holotype CBS H-24380, ex-holotype living culture CBS 146752).

**Figure 4.**
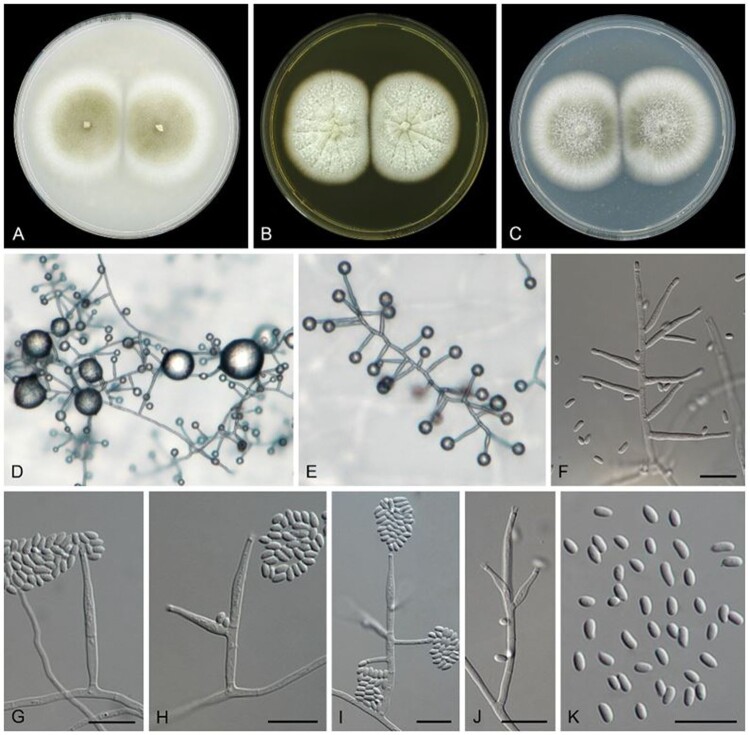
*Phialemoniopsis limonesiae* (ex-type CBS 146752). A. Colonies on OA after 14 d at 25 °C. B. Colonies on MEA. C. Colonies on PDA. D–J. Conidiophores. K. Conidia. Scale bars = 10 μm.

Notes: Phylogenetically, the ex-type strain of *Phialemoniopsis limonesiae* clusters on a single branch which is clearly separated from its closest phylogenetic neighbours *P. curvata* and *P. hongkongensis* (Figure 5) [2,3]. *Phialemoniopsis limonesiae* can be morphologically distinguished from *P. curvata* by its shorter cylindrical or oval conidia, 2.3–4.9 × 1.4–2 μm, while conidia of *P. curvata* are cylindrical or allantoid, 3.5–6(–10) × 1–1.4(–1.7) μm [20]. Besides, *P. limonesiae* can also be distinguished from *P. curvata* by the absence of sporodochium-like conidiomata. *Phialemoniopsis limonesiae* differs from *P. hongkongensis* by producing straight, larger conidia, 2.3–4.9 × 1.4–2 μm, while conidia of *P. hongkongensis* are slightly curved, 2–3 × 1–2 μm [3]. Among all the *Phialemoniopsis* species, *P*. *cornearis*, *P. curvata*, *P*. *ocularis* and *P. limonesiae* grew slowly at 37°C, and *P. limonesiae* sporulated well at 37°C, which is comparable with *P. curvata* [3]; while *P. hongkongensis* and *P. pluriloculosa* showed no growth at 37°C, and the maximum growth temperature of *P. endophytica* was 35°C [2, 3, 4].

Description with culture characteristics after 14 d at 25°C showed (Figure 4) on PDA reaching 55 mm diam, flat, entire margin, floccose, grey olivaceous at centre, white at periphery, reverse concolorous. On OA reaching 50 mm diam, flat, entire margin, dusty, hazel at centre, white at periphery, reverse concolorous. On MEA reaching 52 mm diam, flat, entire margin, radially folded, floccose, dirty white, reverse saffron. The fungus grew at 10°C, 15°C, 20°C, 25°C, 30°C, and 35°C, with optimal growth occurring at 30°C on all three media (up to 75 mm diam in 14 d); slowly grew at 37°C (15 mm diam in 14 d); no growth at 5°C and 40°C. *Mycelium* consisting of branched, septate, hyaline, smooth- and thin-walled hyphae, mostly 1–2(–3.5) μm wide. *Conidiophores* arising from submerged or superficial hyphae, (sub-)erect, simple or branched, bearing 1–2 levels with 2–3 phialides per node, up to 55 μm long, 1.5–2.5 μm wide at the base, hyaline, smooth-walled, with cell walls usually thicker than those of the vegetative hyphae. *Conidiogenous cells* monophialidic, adelophialidic or polyphialidic, terminal, lateral, straight to slightly flexuose, cylindrical, 12–33 μm long, 1.5–2.5 μm wide at the base, with conspicuous collarette and a distinct periclinal thickening at the conidiogenous locus, hyaline, thick- and smooth-walled, adelophialides 4.2–20.2 × 1.1–2.2 μm, polyphialides with up to three conidiogenous loci commonly present. *Conidia* formed in small globose heads at the apex of phialides, ellipsoidal to cylindrical with rounded ends, aseptate, hyaline, thin- and smooth-walled, 2.3–4.9 × 1.4–2 μm, 0–1-guttulate. *Chlamydospores* and sexual morph not observed.

**Figure 5.**
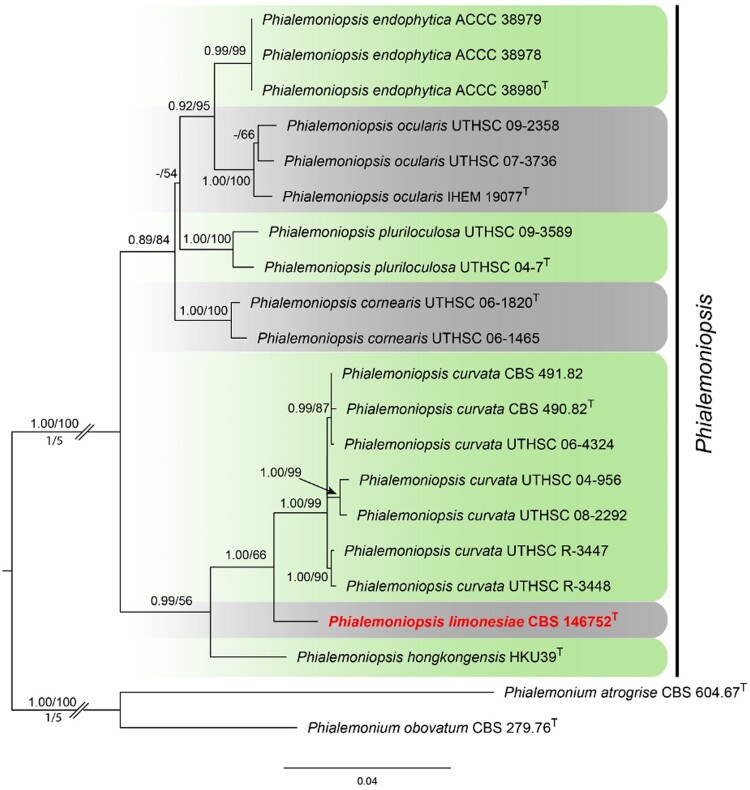
Phylogenetic tree derived from Maximum Likelihood analysis based on combined gene sequences (ITS, LSU, *tub2* and *act*) showing the phylogenetic relationships of the new species *Phialemoniopsis limonesiae* with closely related taxa. The RAxML bootstrap support values (MLBS > 50%) and Bayesian posterior probabilities (BPP > 0.80) are displayed at the nodes (BPP/MLBS). The tree is rooted with *Phialemonium obovatum* (CBS 279.76) and *Phialemonium atrogrise* (CBS 604.67). Ex-type cultures are indicated with a “T” symbol after the accession number.

## Discussion

In this study we described a new opportunistic human pathogen, named *Phialemoniopsis limonesiae*.

Dematiaceous fungi cause opportunistic cutaneous infections most often in solid organ transplant recipients than in AIDS patients [21,22]. In AIDS patients, cryptococcosis, histoplasmosis and *Talaromyces marneffei* infections are more common [23] The genus *Phialemoniopsis* is closely allied to *Phialemonium*, and is widely distributed in air, soil, and plant materials [2]. Different species of *Phialemoniopsis* are associated with infections in humans like endocarditis, endovascular infections, endophthalmitis, meningitis and skin and soft tissue infections [3]. In our case, its most probable origin was Ecuador where our patient travelled prior to symptom development. One possible hypothesis of its emergence could be the use of prolonged antifungal prophylaxis [24].

In our patient, phaeohyphomycosis developed under a treatment of itraconazole capsules at a therapeutic dosage, although the susceptibility testing of the strain showed a low MIC (= 0.06) for itraconazole. However, a low itraconazole blood level (0.81 mg/ml) was confirmed under capsules intake. The lesions improved after the switch to an oral itraconazole solution 200 mg b.i.d. and topical formula consisting of applying an empirical oral itraconazole solution on a dressing during 10 min in addition to standard antiseptics. A higher blood level of oral itraconazole solution compared to the capsules was already described in HIV positive patients treated for oral candidiasis [6]. Our case confirms that the oral solution improves the poor bioavailability due to capsule intake [24]. Standard treatment of known *Phialemoniopsis* spp*.* such as *P*. *curvata* and *P. ocularis* relies on oral itraconazole therapy 100–200 mg daily. Amphotericin B, voriconazole and surgical excision in the case of solitary lesions have also been used [21].

Identification of microbial species as human pathogens is challenging. Particularly moulds and yeasts are commonly isolated from skin on superficial samples. Clear clinical and laboratory criteria need to be used to confirm pathogenicity. We documented each step: repeated samples, biopsy and multiple microbiological analyses. Among these analyses the MALDI-TOF system used in many clinical laboratories for common fungal identification proved inconclusive for this case. To identify rare fungi, the MALDI-TOF system has shown its limitation and its dependency on the quality and robustness of the database used [25].

To conclude, *Phialemoniopsis limonesiae* is a new opportunistic fungus to be added to the growing number of dematiaceous hyphomycete human pathogens, affecting a deeply immunocompromised patient. The clinical presentation as subcutaneous abscesses and nodules on the skin is similar to known *Phialemoniopsis* species in humans.
